# Assessment of Activity Limitations with the Health Assessment Questionnaire Predicts the Need for Support Measures in Patients with Rheumatoid Arthritis: A Multicenter Observational Study

**DOI:** 10.1371/journal.pone.0106749

**Published:** 2014-09-04

**Authors:** Xavier Janssens, Saskia Decuman, Filip De Keyser, the Belgian Rheumatoid Arthritis Disability Assessment study group

**Affiliations:** 1 Department of Rheumatology, Ghent University Hospital, Ghent, Belgium; 2 Department of Internal Medicine, Ghent University, Ghent, Belgium; Center for Rheumatic Diseases, India

## Abstract

**Objective:**

This study investigated whether the Health Assessment Questionnaire (HAQ) can be used as an instrument to assess the need for social support measures that address activity limitations and participation issues in patients with rheumatoid arthritis (RA).

**Methods:**

This multicenter observational study included patients with RA and disease duration of at least one year, consulting their rheumatologist for routine evaluation of disease activity. In the single study visit data on demographics, disease history and current treatment were collected. DAS28 values were collected to evaluate current RA disease activity. Patients were asked to fill out the HAQ and SF-36 questionnaires. Receiver Operator Characteristics (ROC) curves were constructed to evaluate the performance of the HAQ, SF-36 and DAS28 in predicting the need for nine supporting measures available for chronically ill patients in the Belgian social security system. The expert opinion of the treating rheumatologist was used as a reference.

**Results:**

The study included 316 patients with a mean age of 59.8±12.6 years, disease duration of 11.4±9.3 years, mean DAS28 values of 2.83±1.17. Mean HAQ score was 0.95±0.73, mean SF-36 score 56.5±21.3. HAQ scores >1 were observed in 39.4% of patients. The area under the HAQ ROC curve was consistently >0.7 and higher for the HAQ than for SF-36 or DAS28 for all support measures. Rheumatologists on average recommended 3.67 support measures.

**Conclusion:**

The HAQ score was found to be a good predictor of the need for social support measures in patients with RA.

## Introduction

As a chronic inflammatory disease, rheumatoid arthritis (RA) can lead to impairment of physical functioning and mobility, loss of productivity, and difficulties in performing normal daily activities. Patients with RA are confronted with extra costs to compensate for these difficulties, as well as with extra health care costs, often in combination with loss of income [1], [2].

To limit the impact of RA on functioning and quality of life, support measures addressing activity limitations and participation issues are an important complement to the medical treatment. In order to promote equitable allocation of supporting measures and determine the level and type of supporting measures needed, objective and reliable evaluation of the level and area of activity limitations in patients is essential.

The Health Assessment Questionnaire (HAQ) was developed three decades ago at Stanford University by Fries and colleagues as a patient-reported outcome instrument designed to measure health status and health-related quality of life. It is a 20-item questionnaire covering activities of daily living in 8 domains. Respondents indicate for every item whether they can do the activity ‘without any difficulty’, with ‘some’ or ‘much difficulty’ or are ‘unable to do’ the activity. The HAQ yields a disability index (HAQ-DI) score between 0 and 3, where 0 means no disability and 3 represents total dependence on others [3]–[5]. HAQ scores >1 are considered to indicate the presence of disability.

The HAQ is one of the most important patient-reported outcome measures in RA clinical trials, but is also commonly used by rheumatologists in daily practice [6], [7], to assess the functional status of their patients. The HAQ has been translated in many languages [8] and has proven validity and reliability in patients with RA [9], [10].

In the Belgian social security system, nine different social support measures are currently available for persons with chronic daily activity limitations and loss of autonomy, to promote their reintegration and participation. These support measures comprise financial benefits, disease cost reduction measures, tax benefits and social benefits (see Table 1). There is no validated score for allocation of these benefits.

**Table 1:** Social support measures for chronically ill patients in Belgium.

Support measure	Type	Description
Integration allowance	Financial benefit (*<65* years)	To compensate extra costs due to diminished autonomy and to enhance social participation of person with special needs.
Allowance for help to the aged	Financial benefit (*>65* years)	For seniors with chronic health disorders confronted with supplementary costs due to loss of autonomy, in order to enhance their social participation.
Tax reduction	Tax benefit	Income tax exemption for families with one or more disabled persons.
Parking card	Promotion of mobility	For persons with mobility restrictions. Allows parking in parking places reserved for disabled persons and parking without time limit in areas of time restricted parking.
Vehicle tax waiver	Tax benefit	Exemption of car tax for disabled persons
Free public transportation for attendant	Promotion of mobility	Free public transportation access for companions of people with mobility restrictions who can't travel alone
Social telephone rate	Cost reduction	Reduced telephone connection rate, subscription fee and call charges for people with functional limitations.
Social rate utility services	Cost reduction	Exemption of regular allowance and free delivery of a fixed amount of gas and electricity for disabled persons.
Allowance for chronic illness	Financial benefit (*<65* years)	Premium for persons with a chronic disease confronted with high medical costs.

In this study, we investigated whether the HAQ can predict the need for such social support measures in patients with RA, with the expert opinion of the treating rheumatologist as a reference and compared the performance of HAQ, DAS28 and SF-36.

## Patients and Methods

### Ethics Statement

All patients gave written informed consent and the study was approved by the Ethics Committee of the University Hospital Ghent (ref. 2010-094) and the ethical committees of the participating clinical centers.

### Study Design

Patients eligible for inclusion in this multicenter observational study were at least 18 years of age, met the American College of Rheumatology (ACR) classification criteria for RA [11], had a disease duration of at least one year and consulted their rheumatologist for routine half-yearly outpatient evaluation (according to nomenclature number 478030 of the Belgian social security system). No specific exclusion criteria were defined.

To obtain a representative population of patients with RA, participating rheumatologists asked the first eligible patient in every consultation time block to participate in the study. Patients who gave their written informed consent were included in the study and proceeded with the study data collection in the same visit.

Data collected comprised patient demographics, disease history, current treatment, DAS28, HAQ and SF-36 questionnaires. The treating rheumatologists scored the need for each of 9 different supporting measures (Table 1) available for chronically ill patients in the Belgian social security system on a four item categorical scale as: certainly needed, probably needed, probably not needed or certainly not needed. HAQ and SF-36 questionnaires were filled out independently by the patient; the rheumatologists were unaware of the SF-36 or HAQ scores, which were only calculated during data analysis.

### Data analysis and statistics

Data are presented as mean ± standard deviation for normally distributed variables, as median (range) for variables not following a normal distribution or as percentages. Statistical analysis was performed with SPSS version 20 software (IBM Corporation, Armonk, New York, USA). A p-value <0.05 was considered statistically significant.

The Kolmogorov-Smirnov test was used to assess the normal distribution of variables. To compare age groups <65 y versus ≥65 y, unpaired t-test or Mann-Whitney U-test was used for continuous variables, and chi-square test for categorical variables.

To evaluate the performance of the HAQ, SF-36 and DAS28 as tests to predict the need for social support measures by patients with RA, Receiver Operator Characteristics (ROC) curves were constructed and the area under the ROC curve (AUC) was calculated [12], with the recommendation of the treating rheumatologist for all nine support measures (Table 1) as the reference. To this end, the scores ‘certainly needed’ and ‘probably needed’ were considered as a positive recommendation for a particular support measure, unless otherwise stated. For the purpose of constructing the ROC-curve with sensitivity and specificity for the outcome that at least one support measure is considered necessary by the treating rheumatologist, only the score ‘certainly needed’ was considered as a positive score. The relevance of such exercise is that a cut-off for this criterium could define a subpopulation of RA patients who would certainly need at least one support measure and a complimentary subpopulation in which no essential support measures apply.

The AUCs of the ROC curves for HAQ, SF-36 and DAS28 were compared using ANOVA.

The Youden index (sensitivity + specificity – 1) was used to provide a single statistic to capture the performance of the predictive tests [13].

## Results

### Population characteristics

**Figure 1:**
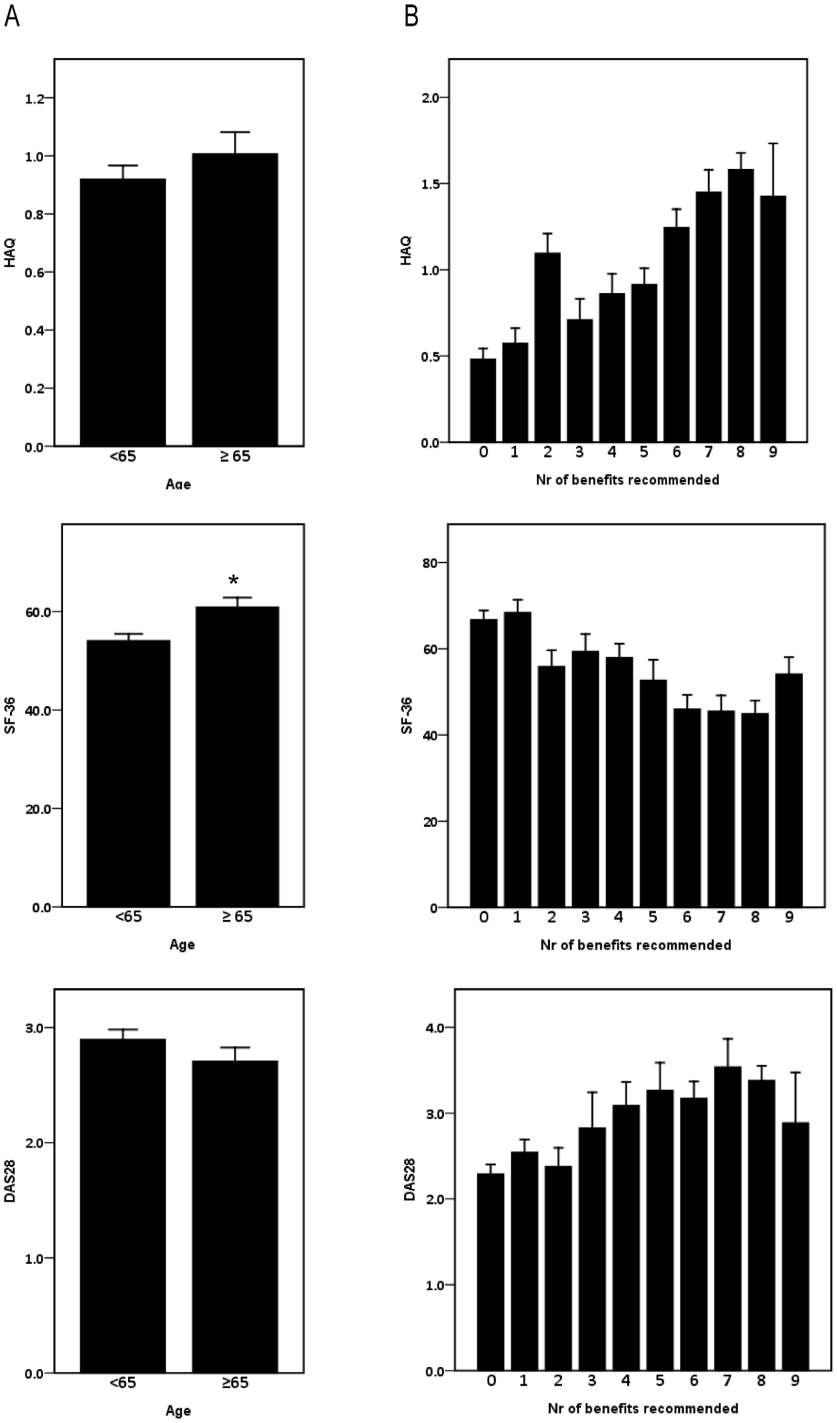
HAQ, SF-36 and DAS 28. A. In function of patient age category. Means for HAQ and DAS28 did not differ between patients below 65 y of age and patients of 65 or older, but patients in the latter age category had significantly higher SF-36 scores *: p<0.05 (t-test, error bars represent standard error of the mean). B. In function of number of benefits recommended by the rheumatologist. (Error bars represent standard error of the mean).

Table 2 summarizes the study population characteristics. The study included 316 patients with RA. Patients were on average 59.8±12.6 years old, with 36.7% (n = 116) of patients aged 65 or older, 69.9% were female. Mean disease duration was 11.4±9.3 years, rheumatoid factor was present in 72.9% of patients and anti-CCP in 58.7%. DAS28 values were 2.83±1.17, HAQ scores were 0.95±0.73 and 39.4% of patients had a HAQ score >1. SF-36 scores were 56.5±21.3, with lower scores on the physical than on the mental subscale, suggesting a larger impact of RA on physical than on mental functioning. HAQ scores and DAS28 values did not differ significantly between patients below 65 years of age and older patients, but patients aged 65 or older had significantly higher SF-36 scores (Figure 1A).

**Table 2:** Population characteristics.

Population characteristics		Overall	<65 y	≥65 y
Nr of patients		316	200	116
% of patients		100	63.3	36.7
Age (y)		59.8±12.6	52.6±9.9	72.31±5.4
Gender ratio (% female)		69.9	73.5	63.8
**RA characteristics**				
Disease duration (y)		11.4±9.3	10.8±8.98	12.2±9.8
RF (% positive)		72.9	71.5	75.2
Anti-CCP (% positive)		58.7	60.5	55.6
Erosions (% positive)		65.5	65.3	66.0
DAS 28		2.83±1.17	2.89±1.16	2.71±1.18
HAQ		0.95±0.73	0.91±0.68	1.01±0.81
	% HAQ >1	39.4	37.0	42.2
SF-36	Total	56.5±21.3	54.0±20.8	60.9±21.6
	MCS	60.6±21.6	58.2±21.4	64.7±21.2
	PCS	49.4±21.4	47.02±20.9	53.6±21.6
**Treatment** (% of patients)				
DMARDs		86.4	86.0	87.1
DMARDs – no biologicals		51.6	45.5	62.1
Biologicals		45.9	52.5	34.5
Corticosteroids		44.6	56.5	46.6
NSAIDs		42.2	44.5	38.4

Data are expressed as mean ± standard error or as percentages. *Abbreviations: CRP: C-reactive protein, DAS28: disease activity score using 28 joint counts, DMARDs: Disease-modifying anti-rheumatic drugs, HAQ: Health Assessment Questionnaire, MCS: mental component summary, NSAIDs: non-steroidal anti-inflammatory drugs, PCS: physical component summary, SF-36: Short Form-36*.

Current RA treatment included DMARDs in 86.4% of patients, 45.9% of patients were on biologicals. Corticosteroids were used by 44.6% and NSAIDs by 42.2% of patients.

### Assessment of the need for social support measures by the rheumatologist

The need for the social support measures listed in Table 1 was scored for every patient by the rheumatologist as certainly or probably needed or certainly or probably not needed. Rheumatologists on average recommended 3.67 support measures per patient.

**Figure 2:**
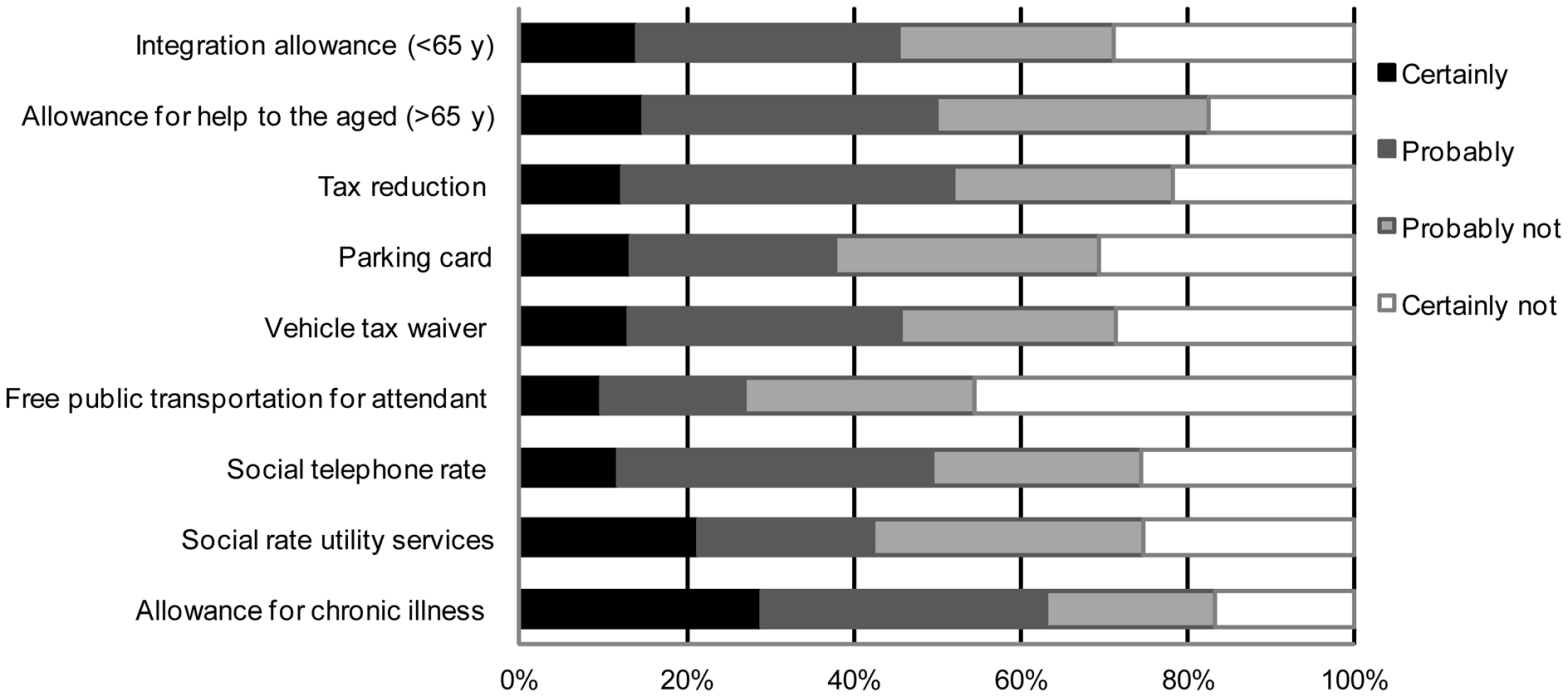
Assessment of need for social support measures by the rheumatologist. Data are expressed as percentage of patients. For support measures with age limits, the patient percentage refers only to the patients in the age category eligible to receive the benefit.

The most frequently recommended support measures were the allowance for chronic illness (62.2%), tax reduction (51.5%) and social telephone rate (49.5%). Integration allowance was recommended for 43.6% of patients <65 years of age, allowance for help to the aged was recommended for 48.6% of patients older than 65. Rheumatologists recommended the social rate for utility services in 42.2% of patients. Vehicle tax waiver, parking card and free public transportation for an attendant were recommended for respectively 45.6%, 37.8% and 26.9% of patients. (Figure 2).

### Predicting the need for social support measures

The number of benefits recommended by the rheumatologist clearly increased with increasing HAQ scores, but not with increasing DAS28 values. Likewise, the number of benefits recommended did not increase with decreasing SF-36 scores (Figure 1B).

In order to assess whether measures of disease activity or functioning can predict the need for social support measures, ROC curve analysis was performed with HAQ, SF-36 and DAS28 for each of the social support measures, with the recommendation of the rheumatologist as reference or gold standard.

**Figure 3:**
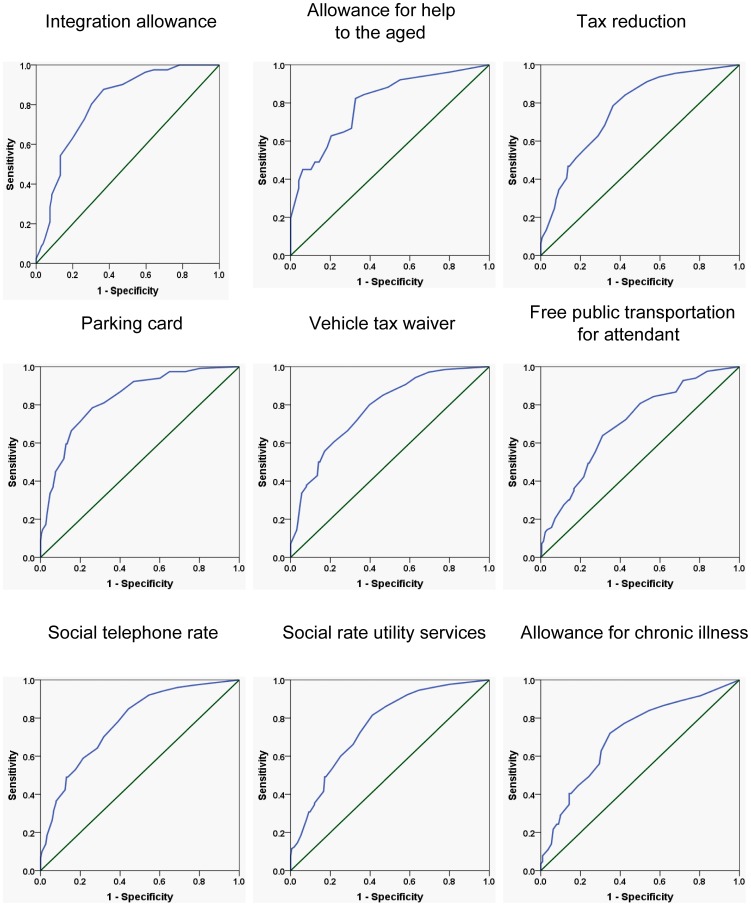
ROC curves for HAQ predicting the need for different social support measures. Receiver operator characteristic analysis (ROC) demonstrates that the Health Assessment Questionnaire (HAQ) performs well as a tool to predict the need for social support measures in patients with RA. The recommendation by the treating rheumatologist was used as reference.

The AUC of these ROC curves are presented in Table 3. For all support measures, AUCs were significantly higher for the HAQ than for either SF-36 or DAS28, with AUC values for HAQ of at least 0.7 for all measures (ANOVA, p = 0.001), indicating that the HAQ score is a valuable predictor of the need for social support measures in patients with RA and is superior in that function to DAS28 and SF-36. Figure 3 shows the ROC curves of the HAQ as predictor for the need for support measures. See File S1 for the raw data for these ROC curves.

**Table 3:** AUC of ROC- curves for HAQ, SF-36 and DAS28.

	HAQ	SF-36	SF-36 PCS	DAS28
Integration allowance (<65 y)	0.806	0.725	0.779	0.695
Allowance for help to the aged (>65 y)	0.798	0.798	0.776	0.785
Tax reduction	0.766	0.727	0.748	0.713
Parking card	0.831	0.728	0.750	0.703
Vehicle tax waiver	0.778	0.710	0.715	0.632
Free public transportation for attendant	0.700	0.642	0.650	0.660
Social telephone rate	0.775	0.708	0.721	0.665
Social rate utility services	0.755	0.735	0.748	0.647
Allowance for chronic illness	0.707	0.639	0.657	0.641

Areas under the curve (AUC) of the ROC curve analysis performed with HAQ, SF-36, SF-36 physical component score (PCS) and DAS28 for the social support measures available in Belgium in order to assess whether these instruments can be used to predict the need for social support measures in patients with RA. The recommendation of the rheumatologist was used as reference.

An additional purpose was to create a ROC-curve with sensitivity and specificity for the outcome that at least one support measure is considered certainly necessary by the treating rheumatologist. In this way, one can propose a cut-off to define a subpopulation of RA patients who would certainly need at least one support measure and a complimentary subpopulation in which no essential support measures apply. Thus, in the latter subpopulation with a score beneath such cut-off, one would not miss any essential support measure. The AUC for such ROC-curve with HAQ, SF-36 and DAS28 was respectively 0.750, 0.698 and 0.656. For the HAQ, the Youden Index for this analysis reached a maximum of 0.391 for a HAQ cut-off of 0.69 (sensitivity 80.8% and specificity 58.3%). In our study population, 133 patients (42.1%) had a HAQ score below 0.69.

No support measures were recommended by the rheumatologist in 24.4% of patients overall and in 43.3% of patients with a HAQ score below 0.69.

## Discussion

Optimal care for patients with RA needs to include support measures to compensate for difficulties in performing daily activities and participation issues. Together, they may alleviate the substantial impact this disease may have.

In this study we evaluated whether disease activity parameters such as DAS28, indicators of functioning such as HAQ, or a measure of health status like SF-36 can predict the need for social support measures, since equitable allocation of benefits requires a reliable measure to evaluate activity limitation.

In this representative, unselected population of patients with longstanding RA, the HAQ score was found to be the best predictor for the need of social support measures, with AUC of the ROC curve of at least 0.7 for all support measures investigated.

Previous studies in the United Kingdom (UK) showed that the HAQ can be used to predict successful application for access to benefits and financial support [14]–[16]. In the UK, 69% of the patients with RA or osteoarthritis of hip or knee and a HAQ score >1.5 who applied were awarded the benefit [15].

In our study, no data were collected on actual applications to receive the social support measures studied, nor on actual allocation of these benefits, so that the results of our study solely represent the expert opinion of the treating rheumatologists on their patients' needs. We did not attempt to calculate HAQ cut-off values for the different support measures investigated, but instead chose to include the raw data of the ROC curves as supplementary data (File S1). Consequently, the data from this study can be used to calculate HAQ cut-offs to suit the sensitivity or specificity limits one wishes to use or to fit the budget available for these benefits.

Based upon our analysis, we propose a HAQ cut-off value of 0.69 to define two RA subpopulations according to whether or not these patients absolutely require at least one of the described support measures. The relevance of this cut-off could be that in case the HAQ score is lower than this cut-off, one could justify not pursuing further efforts for allocation of support measures.

Functioning may be further impaired by factors such as age and comorbidity. It is an important strength of the HAQ to provide a comprehensive assessment of a patient's functioning, not only determined by RA, but also by old age or limitations as a consequence of superimposed comorbidity. The need of support measures will increase with this global loss of functioning and not just with the functional limitations of RA.

We did not collect socioeconomic parameters or educational status in this study. The impact of these parameters on functioning are still controversial: a Swedish cross sectional study reported no influence of socioeconomic factors and education on disability in RA patients [17] but a number of studies have found RA disease activity and severity to be more pronounced in patients with lower socio-economic status [18], [19].

Our findings indicate that it would be feasible to expand the scope of application of the HAQ to include its use as an instrument for allocating access to financial or social support measures or to tax benefits for patients with RA. Such an approach would have the added benefit of reducing the administrative burden involved with benefit allocation by using the results of a test that is widely used and accepted for clinical monitoring of patients with RA.

Additional research is needed to investigate whether the use of HAQ score to determine the need of support can be generalized to other regions, with other types of support measures available, and to determine what the impact would be of using the HAQ for the actual allocation of social support measures. A follow-up study investigating which domains of the HAQ determine best the need for which kind of support measures is currently ongoing.

In summary, the HAQ score was found to be a good predictor (better than DAS28 and SF-36) for the need for social support measures in patients with RA, as assessed by the rheumatologist.

## Supporting Information

File S1 **ROC curve data tables for the HAQ.** Tables with coordinates of the ROC curve analysis investigating the performance of the Health Assessment Questionnaire (HAQ) as an instrument to evaluate the need for social support measures in patients with RA. The expert opinion of the treating rheumatologist on the need for social support measures was used as a reference.
(DOCX)
