# Supplementary material for: Assessment of Activity Limitations with the Health Assessment Questionnaire Predicts the Need for Support Measures in Patients with Rheumatoid Arthritis: A Multicenter Observational Study
Source: PLoS One. 2014 Sep 4;9(9):e106749. doi: 10.1371/journal.pone.0106749 (PMC4154727; doi:10.1371/journal.pone.0106749)
Supplement: File S1 — ROC curve data tables for the HAQ. Tables with coordinates of the ROC curve analysis investigating the performance of the Health Assessment Questionnaire (HAQ) as an instrument to evaluate the need for social support measures in patients with RA. The expert opinion of the treating rheumatologist on the need for social support measures was used as a reference. (DOCX) [file pone.0106749.s001.docx]

File S1: ROC curve data tables for the HAQ

Tables with coordinates of the ROC curve analysis investigating the performance of the Health Assessment Questionnaire (HAQ) as an instrument to evaluate the need for social support measures in patients with RA. The expert opinion of the treating rheumatologist on the need for social support measures was used as a reference.

| Integration allowance (<65y) | | |
| --- | --- | --- |
| Positive if HAQ  Greater Than or Equal To | Sensitivity | 1 - Specificity |
| -1.00000 | 1.000 | 1.000 |
| .06250 | 1.000 | .783 |
| .18750 | .975 | .717 |
| .31250 | .975 | .642 |
| .43750 | .963 | .594 |
| .56250 | .901 | .472 |
| .68750 | .877 | .368 |
| .81250 | .802 | .302 |
| .93750 | .728 | .264 |
| 1.06250 | .630 | .198 |
| 1.18750 | .568 | .151 |
| 1.31250 | .543 | .132 |
| 1.43750 | .444 | .132 |
| 1.53571 | .346 | .085 |
| 1.59821 | .333 | .085 |
| 1.66964 | .284 | .075 |
| 1.73214 | .272 | .075 |
| 1.81250 | .210 | .075 |
| 1.93750 | .123 | .047 |
| 2.06250 | .099 | .038 |
| 2.18750 | .086 | .028 |
| 2.31250 | .062 | .019 |
| 2.43750 | .025 | .000 |
| 2.68750 | .012 | .000 |
| 3.87500 | .000 | .000 |

| Allowance for help to the aged (>65 y) | | |
| --- | --- | --- |
| Positive if HAQ Greater Than or Equal To | Sensitivity | 1 - Specificity |
| -1.00000 | 1.000 | 1.000 |
| .06250 | .961 | .796 |
| .18750 | .941 | .673 |
| .31250 | .922 | .551 |
| .43750 | .882 | .490 |
| .56250 | .843 | .367 |
| .68750 | .824 | .327 |
| .81250 | .667 | .306 |
| .93750 | .647 | .265 |
| 1.06250 | .627 | .204 |
| 1.18750 | .569 | .184 |
| 1.26786 | .490 | .143 |
| 1.33036 | .490 | .122 |
| 1.43750 | .451 | .102 |
| 1.56250 | .451 | .061 |
| 1.68750 | .392 | .041 |
| 1.81250 | .353 | .041 |
| 1.93750 | .275 | .020 |
| 2.06250 | .196 | .000 |
| 2.31250 | .137 | .000 |
| 2.56250 | .098 | .000 |
| 2.68750 | .078 | .000 |
| 2.81250 | .039 | .000 |
| 3.87500 | .000 | .000 |

| Tax reduction | | |
| --- | --- | --- |
| ***Positive if HAQ Greater Than or Equal To*** | ***Sensitivity*** | ***1 - Specificity*** |
| -1.00000 | 1.000 | 1.000 |
| .06250 | .968 | .774 |
| .18750 | .956 | .678 |
| .31250 | .937 | .596 |
| .43750 | .911 | .534 |
| .56250 | .842 | .425 |
| .68750 | .785 | .363 |
| .81250 | .684 | .322 |
| .93750 | .627 | .288 |
| 1.06250 | .563 | .226 |
| 1.18750 | .513 | .178 |
| 1.26786 | .468 | .144 |
| 1.33036 | .468 | .137 |
| 1.43750 | .405 | .130 |
| 1.53571 | .342 | .089 |
| 1.59821 | .335 | .089 |
| 1.66964 | .291 | .075 |
| 1.73214 | .285 | .075 |
| 1.81250 | .247 | .068 |
| 1.93750 | .171 | .041 |
| 2.06250 | .133 | .027 |
| 2.18750 | .108 | .014 |
| 2.31250 | .095 | .007 |
| 2.43750 | .063 | .000 |
| 2.56250 | .044 | .000 |
| 2.68750 | .032 | .000 |
| 2.81250 | .019 | .000 |
| 3.87500 | .000 | .000 |

| Parking Card | | |
| --- | --- | --- |
| ***Positive if HAQ Greater Than or Equal To*** | ***Sensitivity*** | ***1 - Specificity*** |
| -1.00000 | 1.000 | 1.000 |
| .06250 | .991 | .803 |
| .18750 | .974 | .729 |
| .31250 | .974 | .649 |
| .43750 | .940 | .601 |
| .56250 | .922 | .468 |
| .68750 | .871 | .404 |
| .81250 | .810 | .319 |
| .93750 | .784 | .261 |
| 1.06250 | .716 | .202 |
| 1.18750 | .664 | .154 |
| 1.26786 | .595 | .133 |
| 1.33036 | .595 | .128 |
| 1.43750 | .517 | .117 |
| 1.53571 | .448 | .074 |
| 1.59821 | .440 | .074 |
| 1.66964 | .379 | .064 |
| 1.73214 | .371 | .064 |
| 1.81250 | .336 | .048 |
| 1.93750 | .224 | .032 |
| 2.06250 | .172 | .027 |
| 2.18750 | .147 | .011 |
| 2.31250 | .129 | .005 |
| 2.43750 | .086 | .000 |
| 2.56250 | .060 | .000 |
| 2.68750 | .043 | .000 |
| 2.81250 | .026 | .000 |
| 3.87500 | .000 | .000 |

| Vehicle tax waiver | | |
| --- | --- | --- |
| ***Positive if HAQ Greater Than or Equal To*** | ***Sensitivity*** | ***1 - Specificity*** |
| -1.00000 | 1.000 | 1.000 |
| .06250 | .986 | .780 |
| .18750 | .971 | .695 |
| .31250 | .943 | .628 |
| .43750 | .907 | .579 |
| .56250 | .850 | .463 |
| .68750 | .800 | .396 |
| .81250 | .714 | .329 |
| .93750 | .664 | .287 |
| 1.06250 | .607 | .220 |
| 1.18750 | .557 | .171 |
| 1.26786 | .500 | .146 |
| 1.33036 | .500 | .140 |
| 1.43750 | .429 | .134 |
| 1.53571 | .379 | .079 |
| 1.59821 | .371 | .079 |
| 1.66964 | .336 | .055 |
| 1.73214 | .329 | .055 |
| 1.81250 | .286 | .049 |
| 1.93750 | .186 | .037 |
| 2.06250 | .143 | .030 |
| 2.18750 | .114 | .018 |
| 2.31250 | .100 | .012 |
| 2.43750 | .071 | .000 |
| 2.56250 | .050 | .000 |
| 2.68750 | .036 | .000 |
| 2.81250 | .021 | .000 |
| 3.87500 | .000 | .000 |

| Free public transportation for attendant | | |
| --- | --- | --- |
| Positive if HAQ Greater Than or Equal To | Sensitivity | 1 - Specificity |
| -1.00000 | 1.000 | 1.000 |
| .06250 | .976 | .838 |
| .18750 | .940 | .779 |
| .31250 | .928 | .716 |
| .43750 | .867 | .680 |
| .56250 | .843 | .568 |
| .68750 | .807 | .500 |
| .81250 | .723 | .428 |
| .93750 | .687 | .378 |
| 1.06250 | .639 | .311 |
| 1.18750 | .554 | .275 |
| 1.26786 | .494 | .243 |
| 1.33036 | .494 | .239 |
| 1.43750 | .422 | .216 |
| 1.53571 | .361 | .167 |
| 1.59821 | .349 | .167 |
| 1.66964 | .301 | .144 |
| 1.73214 | .301 | .140 |
| 1.81250 | .277 | .117 |
| 1.93750 | .205 | .072 |
| 2.06250 | .157 | .054 |
| 2.18750 | .145 | .032 |
| 2.31250 | .133 | .023 |
| 2.43750 | .084 | .014 |
| 2.56250 | .072 | .005 |
| 2.68750 | .048 | .005 |
| 2.81250 | .024 | .005 |
| 3.87500 | .000 | .000 |
